# Key soil parameters affecting the survival of* Panax notoginseng* under continuous cropping

**DOI:** 10.1038/s41598-021-85171-z

**Published:** 2021-03-11

**Authors:** Wumei Xu, Fengyun Wu, Haoji Wang, Linyan Zhao, Xue Liu, Ping Xiang, Huilin Guan, Lena Q. Ma

**Affiliations:** 1 Yunnan Provincial Observation and Research Station of Soil Degradation and Restoration for Cultivating Plateau Traditional Chinese Medicinal Plants, Yunnan Normal University, Kunming, 650500 China; 2 Yunnan Provincial Renewable Energy Engineering Key Laboratory, Yunnan Normal University, Kunming, 650500 China; 3 Institute of Environmental Remediation and Human Health, Southwest Forestry University, Kunming, 650000 China; 4 Institute of Soil and Water Resources and Environmental Science, College of Environmental and Resource Sciences, Zhejiang University, Hangzhou, 310058 China

**Keywords:** Agroecology, Plant sciences

## Abstract

Negative plant-soil feedbacks lead to the poor growth of *Panax notoginseng* (Sanqi), a well-known herb in Asia and has been used worldwide, under continuous cropping. However, the key soil parameters causing the replant problem are still unclear. Here we conducted a field experiment after 5-year continuous cropping. Sanqi seedlings were cultivated in 7 plots (1.5 m × 2 m), which were randomly assigned along a survival gradient. In total, 13 important soil parameters were measured to understand their relationship with Sanqi’s survival. Pearson correlation analysis showed that 6 soil parameters, including phosphatase, urease, cellulase, bacteria/fungi ratio, available N, and pH, were all correlated with Sanqi’s survival rate (*P* < 0.05). Principal component analysis (PCA) indicated that they explained 61% of the variances based on the first component, with soil pH being closely correlated with other parameters affecting Sanqi’s survival. The optimum pH for Sanqi growth is about 6.5, but the mean soil pH in the study area is 5.27 (4.86–5.68), therefore it is possible to ameliorate the poor growth of Sanqi by increasing soil pH. This study may also help to reduce the replant problem of other crops under continuous cropping since it is widespread in agricultural production.

## Introduction

Perennial plant *Panax notoginseng* (Sanqi) is a well-known herb in Asia, which has been used for thousands of years due to its effects on anti-inflammation and its ability to improve blood circulation^1^. As such, it is a key component of many Chinese medicines, which has been used worldwide and is of significant economic values^2^. The plant is often harvested after 3–7 years of growth. However, its sustainable production has been limited by its poor ability to grow under continuous cropping^3^.

Similar problem is widespread in other crops such as apple, cucumber, strawberry, and potato^4,5^. In ecology, such negative plant-soil feedback is viewed as a type of Janzen–Connell effect^6^. Many factors contribute to the problem including nutrient imbalance^7^, changes in microbial community^8^, and low enzyme activities in the soils^9^. For example, after continuous cropping of Sanqi, Liu et al.^10^ showed P enrichment and K deficiency in soils. Changes in soil-borne microbial communities are also important^11,12^. For instance, Dong et al.^8^ reported the decreased fungal diversity in soils after continuous cropping of Sanqi, which is consistent with the decreased fungal diversity in the rhizospheric soil of root-rot sanqi^13^. In addition, these changes are closely related to soil properties including soil pH and available N^14,15^. Wu et al.^16^ reported that soil pH is an important factor affecting Sanqi’s growth. Wei et al.^17^ showed that over-application of N suppressed bacterial growth, changing the soil microbial community from bacterial-dominant to fungal-dominant.

Soil enzyme is a key parameter that affects the decomposition of soil organic matter, nutrient cycling, and plant growth^18,19^. For example, urease and phosphatase aid in N and P mineralization, while cellulase and phenol oxidase help cellulose and lignin degradation^20^. Soil enzyme activities often decrease under continuous cropping^21^. For example, Lovaisa et al.^5^ reported a significant decline in enzyme activity after 5-year continuous cropping of strawberry. In addition, Sun et al.^22^ showed a decrease in soil enzyme activities with the continued growth of Sanqi, consistent with the significant difference in rhizospheric soil enzyme activities between healthy and diseased Sanqi plants.

Studies have shown that many soil physicochemical factors, enzyme activities, and microbial community are related to the replant problem of Sanqi^7–10,13,17,22^, however, these soil parameters are often closely related and the key soil parameters impacting the survival of Sanqi under continuous cropping are still unclear^23^. For instance, soil pH affects N availability, both affecting plant growth^24^. Therefore, in this study, we conducted a field experiment after 5-year continuous cropping of Sanqi. We determined 13 important parameters including soil pH, plant nutrients, enzyme activities, and bacteria and fungi population. Based on statistical analyses, we correlated them to Sanqi’s survival rates in the field. The information should provide guidance for the sustainable cultivation of Sanqi. In addition, it may help to reduce the replant problem of other crops since it is widespread in agricultural production^25^.

## Materials and methods

### Study site and field experiment

**Figure 1:**
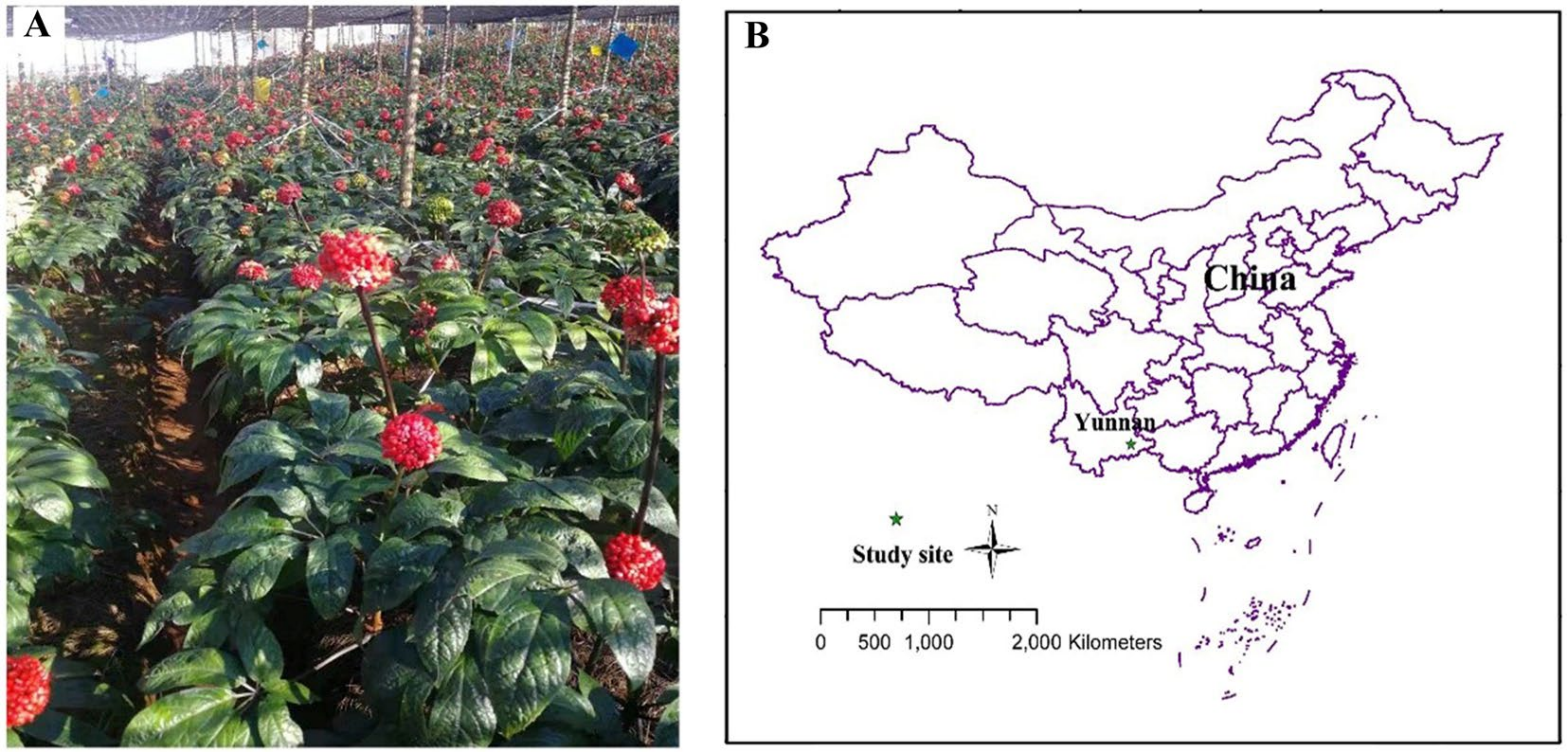
Photo showing the growth of healthy Sanqi (**A**) and the location of the field experiment in Yunnan (**B**). Figure (**B**) was generated by Arcmap 10.2 (https://www.esri.com).

The study was conducted in southwest China at the Miaoxiang Sanqi Science Experiment Station (23° 31′ 44.48″ N, 104° 19′ 13.65″ E) (Fig. 1), which is located at Wenshan, Yunnan, a traditional area for Sanqi production. The annual mean temperature is 16.6℃ and precipitation is 1111 mm^26^. The soil belongs to laterite, which is rich in Fe and Al and often formed under hot and wet tropical climate^8^. The soil pH is 5.94, available N is 38.30 mg kg^−1^, and available P is 6.43 mg kg^−1^, before the cultivation of Sanqi.

Organic fertilizer (OF), which was provided by a local fertilizer company and designed for Sanqi, was applied as the basal fertilizer. OF is produced using livestock manure, the pH is 7.47, available N is ~ 1553 mg kg^−1^, available P is ~ 325 mg kg^−1^, and organic matter content is ~ 1.92%. According to the traditional cultivation of Sanqi, OF was evenly mixed with 0–20 cm topsoil at 0.5 kg m^−2^. Sanqi seedlings of 1-year old were transplanted in February 2017 in the field, which has been growing Sanqi for 5 years. Field management, including weed removal, irrigation, and shading, was the same, and fertilizer was no longer applied after transplanting. Since the soil-borne plant diseases are serious during the rainy season (June to August) partly due to the high soil water content^7^, so its survival rates were investigated in September 2018 after growth of ~ 1.5 years. We randomly selected 7 plots (1.5 m × 2 m) along a survival gradient of Sanqi. The survival rate was calculated as the number of individuals survived/the number of individuals transplanted.

### Soil pH and plant nutrients

To explore the correlations between soil parameters and the survival of Sanqi, soil samples were collected at the same time of survival investigation. For each plot, five soil samples were randomly collected and mixed to make a composite sample. Part of the soil passed through a 2 mm sieve was stored at a 4 °C freezer before microbial analysis and part was air-dried for other analyses. Soil pH was determined by a pH meter (PHS-25) at a soil/water ratio of 1:2.5 after mixing for 30 min. Available soil N was determined by the alkali-hydrolyzed diffusion method and available soil P was determined using the Bray’s method by a spectrophotometer (UV-8000, Yuanxi, China), available soil K, Ca, and Mg were extracted with 1 M CH_3_COONH_4_ solution for 30 min and determined by atomic absorption spectroscopy (AA-7000, Shimadzu, Japan)^27^.

### Soil enzyme activities, and bacteria and fungi population

The activities of 4 important soil enzymes, including phosphatase, urease, cellulase, and polyphenol oxidase, were determined. Briefly, phosphatase was measured using disodium phenyl phosphate colorimetric method, urease the phenol sodium hypochlorite colorimetric method, cellulase the carboxymethyl cellulose method, and polyphenol oxidase the pyrogallol method^28^. The number of bacteria and fungi were determined immediately after the collection of soil samples by counting colony-forming units (CFU). Briefly, 10 g soil was mixed with 90 mL phosphate-buffered saline solution, which was diluted to 10^−6^ (v/v). About 0.1 mL of solution was spread on potato-martin medium to culture the fungus at 37 °C and was spread on beef extract peptone medium to culture bacteria at 25 °C. The CFU was counted when there was no increase in colonies^29^.

### Statistical analyses

The Shapiro–Wilk test was used to evaluate the normal distribution of all parameters, with no variable being rejected (*P* > 0.05). Pearson correlation analysis was used to explore the relations among different soil parameters and Sanqi’s survival rate. Since soil parameters are correlated with each other, we conducted a principal component analysis based on their correlation matrice. All analyses were done using the SPSS 16.0 (SPSS Inc., Chicago, IL, USA) and the R statistical package “Deducer” at *P* < 0.05^30^.

## Results

Soil pH, plant nutrients (N, P, K, Ca and Mg), enzyme activities (phosphatase, urease, cellulase, and polyphenol oxidase), microbial populations (bacteria and fungi), and Sanqi’s survival rates in the 7 plots are shown in Table 1. Briefly, soil pH ranged from 4.86 to 5.68, available N ranged from 96.41 to 134.31 mg kg^−1^, available P ranged from 10.84 to 26.74 mg kg^−1^, available K ranged from 98.14 to 121.41 mg kg^−1^, available Ca ranged from 476.32 to 712.45 mg kg^−1^, and available Mg ranged from 56.23 to 67.24 mg kg^−1^. The activity of phosphatase ranged from 0.18 to 0.44 mg g^−1^ 12 h^−1^, urease ranged from 0.09 to 0.56 mg g^−1^ 24 h^−1^, cellulase ranged from 0.29 to 0.61 mg g^−1^ 24 h^−1^, and polyphenol oxidase ranged from 0.30 to 0.69 mg g^−1^ 2 h^−1^. The CFU of bacteria ranged from 1.32 to 4.17 × 10^6^ g^−1^, fungi ranged from 1.96 to 4.62 × 10^4^ g^−1^, and its ratio (B/F) ranged from 34.85 to 162.26. The data indicate high environmental heterogeneity though field management was the same. Since Sanqi has been growing in the soil for 5 years before the transplanting, its survival rates were low, ranging from 0 to 44.7%, averaging 26%. In other words, 74% of the transferred Sanqi plants did not survive. Without continuous cropping, the survival rate of Sanqi in the experiment station was > 80% in the second growth year generally, the data indicate the serious problem of poor growth of Sanqi under continuous cropping.

**Table 1:** Survival rate of Sanqi and associated soil parameters across the 7 plots.

Soil properties and Sanqi survival rate	Range	Mean ± SD	Plot 1	Plot 2	Plot 3	Plot 4	Plot 5	Plot 6	Plot 7
Survival rate (%)	0.00–44.70	26.43 ± 14.02	0.00	21.20	24.60	26.80	32.10	35.60	44.70
pH	4.86–5.68	5.27 ± 0.28	4.86	5.09	5.17	5.37	5.20	5.54	5.68
Available N (mg kg^−1^)	96.41–134.31	121.03 ± 12.04	96.41	120.46	118.56	124.02	124.89	128.57	134.31
Available P (mg kg^−1^)	10.84–26.74	19.34 ± 5.42	10.84	16.78	24.21	15.47	26.74	20.21	21.14
Available K (mg kg^−1^)	98.14–121.41	112.01 ± 7.51	98.14	110.96	118.73	110.74	121.41	109.85	114.21
Available Ca (mg kg^−1^)	476.32–712.45	581.81 ± 112.02	476.32	477.16	498.97	689.76	519.15	712.45	698.87
Available Mg (mg kg^−1^)	56.23–67.24	61.15 ± 3.76	56.23	64.87	59.71	61.41	67.24	58.74	59.86
Phosphatase (mg g^−1^ 12 h^−1^)	0.18–0.44	0.33 ± 0.09	0.18	0.24	0.34	0.36	0.31	0.42	0.44
Urease (mg g^−1^ 24 h^−1^)	0.09–0.56	0.36 ± 0.15	0.09	0.28	0.35	0.39	0.37	0.47	0.56
Cellulase (mg g^−1^ 24 h^−1^)	0.29–0.61	0.42 ± 0.12	0.30	0.29	0.39	0.52	0.37	0.49	0.61
Polyphenol oxidase (mg g^−1^ 2 h^−1^)	0.30–0.69	0.45 ± 0.13	0.69	0.30	0.38	0.40	0.41	0.51	0.49
Bacteria (× 10^6^ CFU g^−1^)	1.32–4.17	2.44 ± 1.08	1.61	1.32	1.58	2.89	2.09	3.42	4.17
Fungi (× 10^4^ CFU g^−1^)	1.96–4.62	2.71 ± 0.94	4.62	2.12	2.07	2.43	1.96	3.18	2.57
Ratio of bacteria to fungi	34.85–162.26	95.54 ± 41.68	34.85	62.26	76.33	118.93	106.63	107.55	162.26

### Correlation of Sanqi’s survival rates with 13 soil parameters

**Figure 2:**
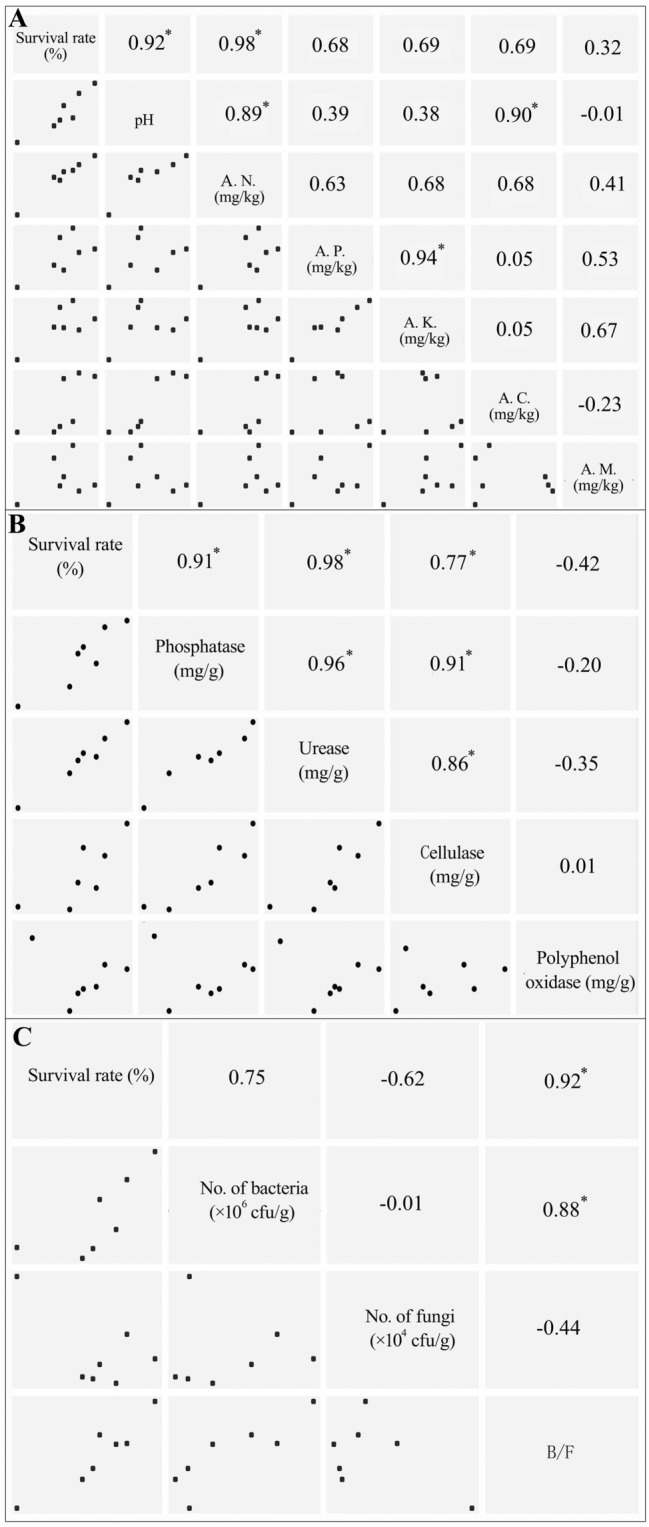
Correlations between Sanqi’s survival rate and soil pH and plant nutrients (**A**), soil enzyme activities (**B**), and soil bacteria and fungi population (**C**) based on Pearson correlation coefficient with *P < 0.05. The figure was generated by function ggcorplot in the R package “Deducer”^30^. *B/F *ratio of bacteria to fungi, *AN *available N, *AP *available P, *AK *available K, *AC *available Ca, *AM *available Mg.

Pearson correlation analysis indicated soil pH and available N were positively correlated with Sanqi’s survival rate (r = 0.92–0.98, *P* < 0.01), while correlations for available P, K, Ca, or Mg were insignificant. Besides, soil pH was highly correlated to available N (r = 0.89, *P* < 0.01) (Fig. 2). While the activities of phosphatase, urease and cellulase were correlated with Sanqi’s survival rate (r = 0.91, 0.98, and 0.77, *P* < 0.04), the correlation for polyphenol oxidase was insignificant (*P* > 0.05). In addition, the enzymes correlated with Sanqi’s survival rate were also correlated with each other (Fig. 2).

The ratios of soil bacteria/fungi (B/F) were correlated with Sanqi’s survival rates (r = 0.94, *P* < 0.01; Fig. 2). However, the correlation between Sanqi’s survival rates and soil bacteria or fungi population was insignificant (r = 0.75, *P* = 0.06; r = − 0.62, *P* = 0.14). The number of soil bacteria was positively correlated with bacteria/fungi ratio while fungi was not, indicating the ratio was mainly determined by soil bacteria (Fig. 2).

### Principal component and Pearson correlation analyses of 13 soil parameters

**Figure 3:**
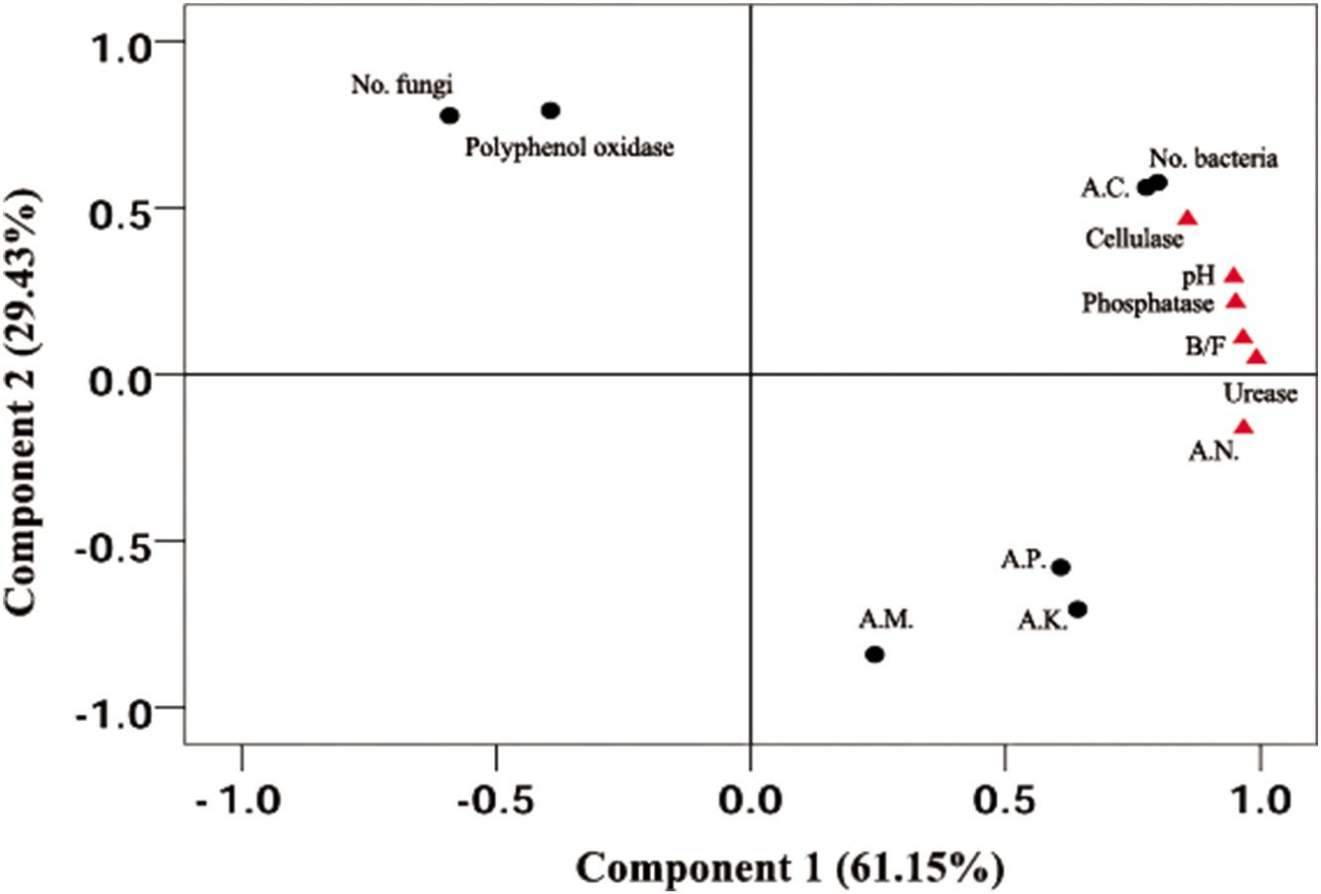
Principal component analysis of 13 soil parameters in this study, with factors marked with triangle indicating significant correlation with Sanqi’s survival rate. *B/F *ratio of bacteria to fungi, *AN *available N, *AP *available P, *AK *available K, *AC *available Ca, *AM *available Mg.

Principal component analysis indicated that the first component (PC1), consisting of soil pH, available N, available Ca, phosphatase, urease, cellulase, bacteria population and B/F ratio, explained 61.15% of the variances. All parameters were correlated with Sanqi’s survival rate excluding available Ca and bacteria population (Fig. 3), indicating the intrinsic relevance of the soil parameters related to the survival of Sanqi. Further, Pearson correlation analysis indicated that soil pH was highly correlated with available N, B/F, and the activities of urease, cellulase and phosphatase (r = 0.92–0.98, *P* < 0.01), indicated that soil pH could be a key parameter for the continuous cropping of Sanqi.

## Discussions

### Soil pH and available N were correlated with Sanqi’s survival rate

Our study showed that soil pH and available N were positively correlated with Sanqi’s survival rate (Table 1; Fig. 2), similar results were also observed by Wu et al.^16^. Soil pH plays an important role in controlling Sanqi’s growth under continuous cropping. For example, Yang et al*.*^31^ showed that steaming combined with biochar application increased soil pH as well as the survival rate of Sanqi under continuous cropping. Meanwhile, Tan et al.^32^ reported that the continuous cropping of Sanqi affected the community structure and diversity of soil bacteria, which were influenced by soil pH. This is because Sanqi roots secrete organic acids to the rhizosphere, causing soil acidification^33^. This has been confirmed by Sun et al.^22^ who showed soil pH declined 1.6 from 7.0 to 5.4 after 3-year continuous cropping. Sanqi prefers to grow in slightly acid soil, with the optimum pH being ~ 6.5^34^. In this study, after 5-year continuous cropping, the soil pH in the plots was more acidic at 5.27 ± 0.28 (Table 1). Therefore, increasing soil pH to ~ 6.5 may benefit Sanqi’s growth and improve its survival rate under continuous cropping.

The positive correlation between soil available N and Sanqi’s survival rate was not unexpected. Wei et al.^17^ showed that the addition of 225 kg N ha^−1^ decreased its disease index and promoted Sanqi’s growth. However, high N addition at 450 kg N ha^−1^ promoted pathogen accumulation in soil. The content of available N in the 7 plots were 96.41–134.31 mg kg^−1^, much lower than the average level at 213 mg kg^−1^ of the main production area for Sanqi^35^. Therefore, slightly increasing N may benefit Sanqi’s growth and help guard against soil-borne diseases. However, no correlations between Sanqi’s survival rate and other plant nutrients including P, K, Ca, and Mg were found (Fig. 2), indicating that these soil parameters could be not important for the survival of Sanqi under continuous cropping.

### Soil enzyme activities and bacteria/fungi ratios were correlated with Sanqi’s survival rate

Besides soil pH and available N, the activities of phosphatase, urease, and cellulase were also correlated with Sanqi’s survival rates (Table 1; Fig. 2). Enzymes are key soil parameters, which play important biochemical functions in the decomposition of organic matter and mineralization of plant nutrients like N and P^18^. For example, phosphatase helps to mineralize organic P, which responds to changes in soil management^19^. Decline in soil enzyme activities have been observed for crops after continuous cropping including strawberry, cucumber, potato, cotton, and Sanqi, partly due to the altered soil properties and microbial communities^5,22,36–38^. For instance, Sun et al.^22^ showed that the activities of phosphatase and urease in the soil decreased 69 and 92% after 3 year of continuous cropping of Sanqi. The positive correlations between the activities of cellulase, urease, phosphatase, and Sanqi’s survival rate indicate that the accelerated C, N, and P cycling with increased soil enzyme activities could benefit the survival of Sanqi under continuous cropping^39^. Also, these soil enzymes can be used as soil parameters affecting Sanqi growth under continuous cropping^40^.

Soil enzyme activities were closely related to soil microbial populations. For example, the number of soil bacteria was highly correlated with the activities of phosphatase, urease, and cellulase (r = 0.82–0.94, *P* < 0.05), while the number of soil fungi was highly correlated with the activity of polyphenol oxidase (r = 0.93, *P* < 0.01). The data indicate that the soil enzyme activities under continuous cropping of Sanqi were affected by soil microorganisms.

Besides soil pH, available N, and three soil enzyme activities, the ratios of soil bacteria/fungi were correlated with Sanqi’s survival rates (r = 0.94, *P* < 0.01; Table 1; Fig. 2). While the ratios are affected by soil nutrient status and pH, but the changes in the ratios also affect litter decomposition, nutrient cycling, and crop growth^41,42^. For example, Liu et al.^43^ showed that soil fumigation and application of bio-organic fertilizer caused a more bacterial-dominant microbial community, which reduced plant diseases and increased crop yields under the continuous cropping of potato. For Sanqi, recent studies indicated that continuous cropping cause a shift from a more bacterial- to more fungal-dominant microbial community^44^. Besides, the high N input led to fungal-dominant microbial community, which exacerbated the poor growth of Sanqi^17^. In this study, though bacterial or fungal population did not correlate with Sanqi’s survival rate, the bacteria/fungi ratio was positively correlated (Fig. 2). The data suggest a bacterial-dominant soil microbial community may benefit Sanqi’s growth.

### Soil pH as a key parameter for Sanqi’s survival under continuous cropping

**Figure 4:**
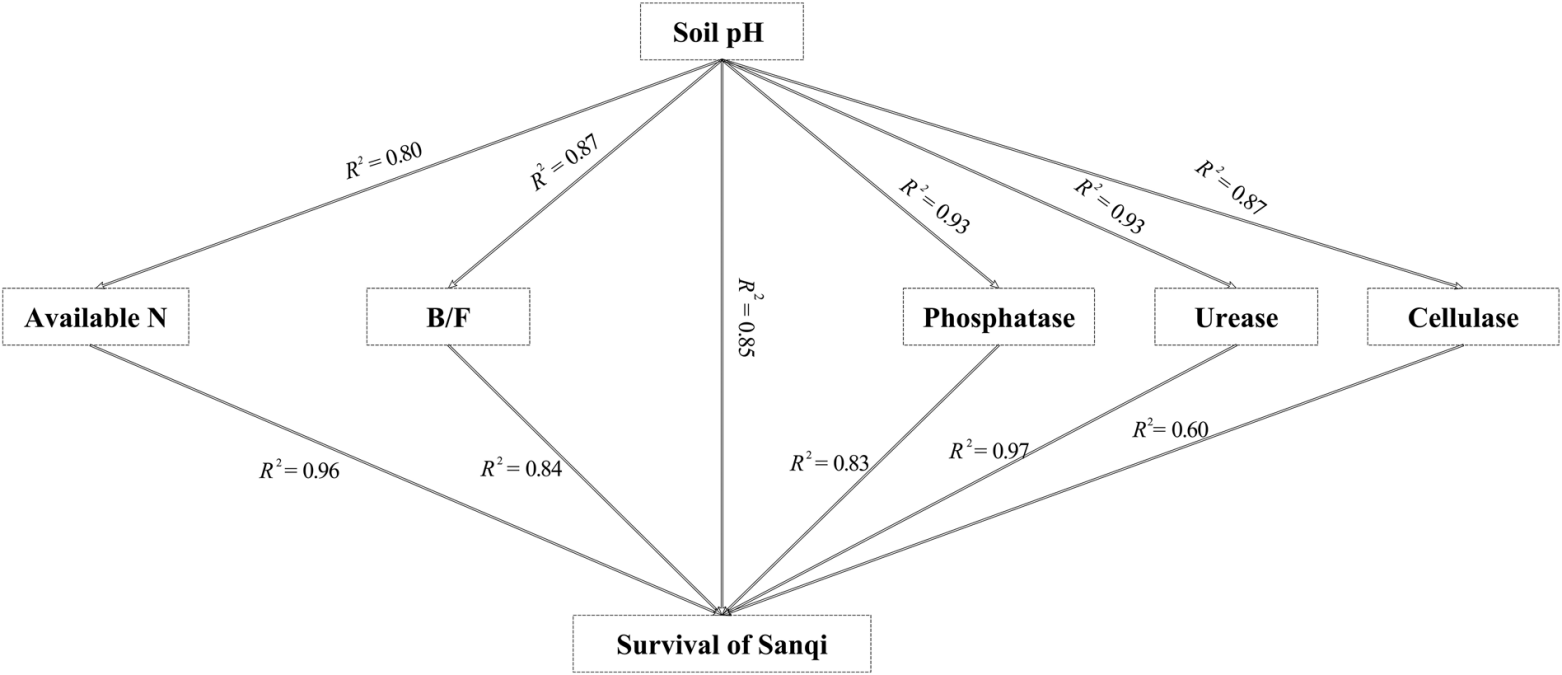
Correlations of soil pH with 5 key soil parameters impacting Sanqi’s survival rates. *R*^2^ is the coefficient of determination in linear regression analysis, with *P* < 0.05.

Soil pH is an important chemical indicator of soil health, which has a major impact on soil biochemical functions^45^. Correlation and principal component analyses indicated that soil pH was a key soil parameter for the continuous cropping of Sanqi (Figs. 3, 4). In addition, soil pH was also correlated with other soil parameters (Fig. 4). For example, soil pH impacts enzyme activity, with each enzyme having optimal activities over certain pH values^46^. Further, soil enzyme activity is affected by microbial activities^47^, which is again related to soil pH^48,49^. Soil pH has a significant impact on bacteria/fungi ratios as fungi are more acid-tolerant than bacteria^41^. In this study, we observed a shift from a bacterial-dominant to a fungal-dominant microbial community with decreasing soil pH (Fig. 4), which may increase pathogen population and impact Sanqi’s growth^17^. A recent study regarding ginseng, which is also a famous medicinal plant and closely related to Sanqi, indicated that soil pH decreased 0.73, from 5.71 to 4.98, under 3-year continuous cropping significantly inhibited the growth of toxin-degrading bacteria and increased the death rate of ginseng^50^. Also, soil pH decreased 0.9, from 7.60 to 6.70, after the continuous cropping of strawberry for 7 years, which caused the accumulation of four phenolic acids (i.e., *p*-hydroxybenzoic acid, ferulic acid, cinnamic acid, and *p*-coumaric acid) and the poor growth of strawberry^51^. Urease is related to N mineralization^20^, in this study, urease activity was highly correlated with soil available N (r = 0.96, *P* < 0.01). The data suggest that increased urease activity with soil pH may promote N mineralization, thereby increasing soil N availability and the survival rate of Sanqi under continuous cropping (Fig. 4).

Sanqi is a well-known herb in Asia and has been used worldwide, however, its sustainable production was largely limited due to its poor growth under continuous cropping and the key soil parameters leading to the replant problem are still unclear. Our study showed that many soil parameters including soil phosphatase, urease, cellulase activity, bacteria/fungi ratio, available N, and pH were all correlated with Sanqi’s survival. In addition, correlation and principal component analysis indicated that soil pH correlated with other soil parameters related to Sanqi’s survival under continuous cropping, alluding to the possible mechanisms. Therefore, soil pH was a key soil parameter for the successful growth of Sanqi. Increasing soil pH to ~ 6.5 may ameliorate the poor growth of Sanqi under continuous cropping. However, as this study was based on the field investigation, more experiments are needed to verify the complex mechanisms leading to the replant problem of Sanqi.
